# The lectin ArtinM activates RBL-2H3 mast cells without inducing degranulation

**DOI:** 10.1371/journal.pone.0230633

**Published:** 2020-03-24

**Authors:** Patricia A. A. Buranello, Valéria C. Barbosa-Lorenzi, Marcelo R. Pinto, Gabriela Pereira-da-Silva, Maria Cristina R. A. Barreira, Maria Célia Jamur, Constance Oliver

**Affiliations:** 1 Department of Cell and Molecular Biology and Pathogenic Bioagents, Ribeirão Preto Medical School, University of São Paulo, Ribeirão Preto, São Paulo, Brazil; 2 Department of Maternal-Infant Nursing and Public Health, Escola de Enfermagem de Ribeirão Preto, University of São Paulo, Ribeirão Preto, São Paulo, Brazil; Universidade Federal do Rio de Janeiro, BRAZIL

## Abstract

Mast cells are connective tissue resident cells with morphological and functional characteristics that contribute to their role in allergic and inflammatory processes, host defense and maintenance of tissue homeostasis. Mast cell activation results in the release of pro-inflammatory mediators which are largely responsible for the physiological functions of mast cells. The lectin ArtinM, extracted from *Artocarpus heterophyllus* (jackfruit), binds to D-manose, thus inducing degranulation of mast cells. ArtinM has several immunomodulatory properties including acceleration of wound healing, and induction of cytokine release. The aim of the present study was to investigate the role of ArtinM in the activation and proliferation of mast cells. The rat mast cell line RBL-2H3 was used throughout this study. At a low concentration (0.25μg/mL), ArtinM induced mast cell activation and the release of IL-6 without stimulating the release of pre-formed or newly formed mediators. Additionally, when the cells were activated by ArtinM protein tyrosine phosphorylation was stimulated. The low concentration of ArtinM also activated the transcription factor NFkB, but not NFAT. ArtinM also affected the cell cycle and stimulated cell proliferation. Therefore, ArtinM may have therapeutic applications by modulating immune responses due to its ability to activate mast cells and promote the release of newly synthesized mediators. Additionally, ArtinM could have beneficial effects at low concentrations without degranulating mast cells and inducing allergic reactions.

## Introduction

ArtinM, also known as KM+ or Artocarpin [1], is a mannose binding lectin that is extracted from the seeds of *Artocarpus heterophyllus* (jackfruit). ArtinM is 64 kDa homotetramer formed by the association of 16 kDa non-glycosylated subunits [2, 3]. ArtinM has a high specificity for Manα1–3(Manα1–6)Man, the trimannoside core of *N*-glycans [4]. This specificity of ArtinM enables the lectin to bind to diverse glycoproteins on the cell surface, thus modifying cellular function. Several immunomodulatory properties have been described for ArtinM, including increased IL-12 production that then leads to a Th1 immune response against intracellular pathogens. Additionally, ArtinM is also known to interact with macrophages, neutrophils, dendritic cells and mast cells and may thus modulate inflammatory processes [5–7].

It has previously been shown that ArtinM can act on mast cells, degranulating them in an IgE-independent manner [8, 9] and inducing the recruitment of immature mast cells from the bone marrow to the peritoneal cavity [10]. The role of mast cells in IgE-mediated allergic and inflammatory processes is well established [11]. Mast cells are also known to play a role in host response to pathogens, participate in autoimmune diseases and in wound healing [12–14]. The main pathway for mast cell activation is through the high affinity receptor for IgE (FcɛRI) whose activation culminates in a rapid release of preformed mediators and newly formed lipid mediators as well as the subsequent release of newly synthesized mediators such as cytokines and growth factors [15, 16]. However recent data has shown that low antigen concentrations (0.1ng/mL) can activate FcɛRI leading to activation of the transcription factor NFAT without inducing mast cell degranulation [17]. RBL-2H3 mast cells can also be partially activated without inducing degranulation by binding mAbAA4 to mast cell specific gangliosides on the cell surface [18, 19].

Since ArtinM has possible therapeutic applications, it was of interest to investigate the effects of low concentrations of ArtinM, on mast cells. The ability of a low concentration (0.25μg/mL) of ArtinM to stimulate RBL-2H3 mast cells *in vitro* and to induce liberation of the various classes of mediators was examined. The mode of mast cell activation was also investigated. Finally, the effect of the low concentration of ArtinM on mast cell proliferation was characterized. Although ArtinM in high concentrations results in mast cell degranulation [8, 10, 20], the results of the present study show that at a low concentration ArtinM does not degranulate mast cells, but does activate mast cells to release cytokines that are important in modulating inflammatory processes [21, 22].

## Materials and methods

### Cells

The rat mast cell line, RBL-2H3, was grown as monolayers as previously described [23]. The VB9 (RBL-2H3 NFAT-GFP) cells and the NFκB2 (RBL-2H3 NFκB-GFP) cells were cultured at 37 °C in Dulbeco’s Modified Eagle’s Medium (DMEM) (Thermo Fisher Scientific, Invitrogen, Carlsbad, CA) containing 10% fetal bovine serum (FBS) (Millipore Sigma, St. Louis, MO), and 0.4 mg/mL geneticin (Millipore Sigma) in a humidified atmosphere of 5% CO_2_ in air. [17]kindly provided by Reuben P. Siraganian, NIDCR, NIH, Bethesda, MD) Cell viability was determined by trypan blue exclusion. Cells were suspended in PBS 1: 1 with 0.4% trypan blue (Thermo Fisher, Invitrogen) solution and the percentage of viable cells determined using a Neubauer camera. Cell viability was between 98%-99% for all experimental conditions.

### ArtinM preparation

ArtinM was purified as previously described [2, 3] from the saline extract of *Artocarpus heterophyllus* (jackfruit) seeds through affinity chromatography with immobilized carbohydrate columns. After purification, the ArtinM aliquots were passed over a Detoxi-Gel^™^ Endotoxin Removing Gel column (Thermo Scientific, Waltham, MA) to remove LPS and other endotoxins.

### Mediator release assays

#### Histamine

Histamine assays were performed as follows. Briefly, the histamine was extracted into n-butanol from alkalinized perchloric acid tissue extracts. The histamine was returned to an aqueous solution and condensed with o-phthalaldehyde (Millipore-Sigma) to yield a fluorescent product. The intensity of fluorescence was measured using the Histamine Module for the Trilogy Laboratory Fluorometer (Turner Designs, Inc., Sunnyvale, CA).

#### β-Hexosaminidase activity

3.0×10^4^ cells/well were plated in 96 well tissue culture plates (Corning Life Sciences, Corning, NY) and cultured overnight. The cells were then incubated with 0.25, 2.5 and 25 μg/mL of ArtinM in Tyrode’s buffer (137 mM NaCl; 2.7 mM KCl; 12 mM NaHCO_3_; 0.37 mM NaH_2_PO_4_; 0.1 mM MgCl_2_; 1.3 mM CaCl_2_ and 10 mM HEPES, pH 7.3) supplemented with 0.01% gelatin (Millipore Sigma) and 0.1% BSA (Millipore Sigma) for 45 min. As a positive control, cells were stimulated via FcεRI. The cells were sensitized with a 1:5000 dilution of mouse IgE anti-TNP ascites fluid in the culture medium and incubated for 16 h and then stimulated with 50ng/mL of DNP_48_-HSA (Millipore Sigma), in Tyrode’s buffer for 45 min. As a negative control, cells were sensitized with IgE, but not stimulated with DNP_48_-HSA. Degranulation of the RBL-2H3 cells was assessed by measuring the release of β-hexosaminidase activity [24].

#### Detection of newly formed lipid mediators

1x10^5^ cells were cultured for 4 h with 0.25, 2.5 or 25 μg/ml ArtinM diluted in DMEM. The amount of LTB_4_, and LTC_4_ released into the culture supernatants were analyzed using EIA kits (Cayman Chemical, Ann Arbor, MI).

#### Cytokine detection

1x10^5^ cells were cultured for 12 h with 0.25, 2.5 or 25 μg/mL ArtinM diluted in DMEM. The concentration of IL-6, IL-4 and MCP-1 in the cell culture supernatants was measured by ELISA (OPTEIA^™^ Rat IL-6 ELISA kit II—BD Biosciences; OPTEIA^™^ Rat IL-4 ELISA kit II—BD Biosciences), according to the manufacturer’s instructions. Cytokine concentrations (pg/ml) were determined from the appropriate standard curve.

#### Scanning electron microscopy

RBL-2H3 cells (2x10^4^ cells/well) were plated on glass coverslips placed in the wells of 24-well plates (Corning Life Sciences) and cultured overnight. The cells were then incubated with 0.25, 2.5 and 25 μg/mL of ArtinM as described above for β-hexosaminidase activity. After 45 min, the cells were washed in warm (37 °C) PBS, fixed in 2% glutaraldehyde (Electron Microscopy Sciences, Hatfield, PA) in warm PBS (containing 0.90 mM Ca^2+^ and 0.50 mM Mg^2+^) for 2 h at room temperature. After fixation, cells were washed twice with 0.1 M cacodylate buffer, pH 7.4 and post-fixed in 1% OsO_4_ (Electron Microscopy Sciences) in Milli-Q water for 2 h at room temperature. Subsequently, cells were washed in Milli-Q water and incubated in a saturated solution of thiocarbohydrazide (Electron Microscopy Sciences) for 10 min at room temperature. After this, the samples were washed five times with Milli-Q water and then incubated in 1% OsO_4_ in Milli-Q water. The cells were then dehydrated in increasing ethanol solutions (30, 50, 70, 90 and 100%) and critically point dried using CO_2_ (BAL-TEC-DPC-030 Critical Point Dryer, Balzers, Germany). Each coverslip was then attached with silver paint (Electron Microscopy Sciences) to an aluminum stub and covered with gold in a sputter coater (BAL-TEC-CPD-050 Sputter Coater, Balzers, Germany). The samples were then observed in a JEOL JSM-6610 LV (Tokyo, Japan) scanning electron microscope.

### Tyrosine phosphorylation assay

Tyrosine phosphorylation was examined as previously described [25]. 2x10^5^ cells/well were plated in 24 well plates and cultured overnight. The cells were then incubated with 0.25 μg/mL of ArtinM in DMEM. As a positive control, cells were sensitized with a 1:5000 dilution of mouse IgE anti-TNP ascites fluid in the culture medium and incubated for 16 h and then stimulated with 50 ng/mL of DNP_48_-HSA (Millipore Sigma) in DMEM. Cells that were not sensitized with IgE and stimulated with DNP_48_-HSA served as a negative control. At 5, 10 and 15 min after addition of ArtinM or DNP_48_-HSA, cell monolayers were washed twice with ice cold PBS containing 1mM Na_3_VO_4_ (Millipore Sigma) and immediately lysed with hot sample buffer (160 mM Tris-HCl, pH 6.8, 4% SDS, 20% glycerol, 0.005% bromophenol blue, 2 mM Na_3_VO_4_ and 1% protease inhibitor cocktail; Millipore Sigma). The cell lysates were boiled for 15 minutes, and the proteins were separated electrophoretically on 10% polyacrylamide gels under reducing conditions (2% β-mercaptoethanol; Millipore Sigma) and transferred to Hybond membranes (GE-Healthcare, Marlborough, MA). After transfer, the membranes were blocked for 16 h at 4°C in TTBS (0.05 M Tris-HCl, 0.15 M NaCl, pH 7.5, and 0.05% Tween 20) containing 4% BSA. After blocking, the membranes were incubated for 1h at room temperature with mAb PY99 (Santa Cruz Biotechnology, Dallas, TX) diluted 1:500 in TTBS. After washing five times with TTBS the membranes were incubated with donkey anti mouse IgG conjugated to HRP (Jackson ImmunoResearch, West Grove, PA) diluted 1:20,000 in TTBS for 1 h at room temperature. Membranes were then washed in TTBS ten times and developed using chemiluminescence (ECL-GE Healthcare). Optical density was determined using Photoshop 7.0 (Adobe Systems Incorporated, San Jose, CA).

### Transcription factor activation

A GFP reporter was used as a marker of NFAT and NFκB activation. The VB9 and NFκB -GFP cells, kindly provided by Reuben P. Siraganian, NIDCR, NIH, Bethesda, MD, were produced as previously described [17]. The VB9 cells were produced by transfecting RBL-2H3 cells with linearized plasmid containing three tandem NFAT binding sites fused to GFP. To produce the NFκB2 cells, RBL-2H3 cells were transfected with four tandem NFκB binding sites upstream of the promoter mCMV that regulates the expression of GFP dependent on the activation of NFκB. Transfected cells were selected by geneticin (G-418) and then cloned to select cells that had maximum GFP fluorescence response after stimulation. 2.5×10^4^ cells/well were plated in 24 well tissue culture plates (Corning Life Sciences). VB9 and NFκB2 cells were stimulated with ArtinM and analyzed 5 h (NFκB) or 20 h (NFAT) after stimulation. These periods of stimulation were found to result in maximal GFP expression. For positive controls, cells were sensitized with a 1:5000 dilution of mouse IgE anti-TNP ascites fluid in the culture medium and incubated for 16 h and then stimulated with 50ng/mL of DNP_48_-HSA (Millipore Sigma). The fluorescence levels were measured using a Guava Personal Cell Analysis-96 System and data were processed using Guava InCyte Software (Millipore Sigma).

### Cell proliferation assays

#### Cell cycle analysis

RBL-2H3 cells were synchronized in G1 using a double thymidine block. Cells were incubated with 2 mM thymidine for 18 h and then released into thymidine-free medium with 2 mM deoxycytidine for 9 h. The cells were again incubated with thymidine for 18 h and released into thymidine-free medium with deoxycytidine for 9 h. The cells were then washed twice with culture medium and 0.25 μg/mL of ArtinM was added to each well. At 0, 3, 6, 9 and 12 h after addition of ArtinM, the cells were stained with propidium iodide and analyzed by flow cytometry as described in section 5.6.

#### Phosphohistone H3 immunostaining

0.7x10^6^ cells/well were plated in 6 well tissue culture plates (Corning Life Sciences) and synchronized using a double thymidine block as described in section 5.7.1. The cells were then cultured for 5 h in the presence of 0.25 μg/mL of ArtinM. The cells were harvested with trypsin–EDTA (Thermo Fisher Scientific, Invitrogen) and washed by centrifugation (127 x *g*/5min) in PBS. The cells were fixed for 20 min with 2% formaldehyde (Electron Microscopy Sciences) in PBS and permeabilized with 0.1% saponin (Millipore Sigma). Cells were blocked for 30 min at room temperature in PBS containing 1% BSA and 5 μg/mL donkey IgG (Jackson ImmunoResearch). The cells were then incubated with 2 μg/mL anti-phosphohistone H3 (Millipore Sigma) diluted in PBS for 1 h at RT. The cells were washed 5 times in PBS and incubated for 30 min at room temperature with goat anti-rabbit IgG conjugated to Alexa 488 (Thermo Fisher Scientific, Molecular Probes) diluted 1:300 in PBS. After rinsing, the cells were analyzed by flow cytometry as described in section 5.6.

#### Crystal violet

2.5×10^4^ cells/well were plated in 24 well tissue culture plates (Corning Life Sciences) and cultured overnight. The cells were then cultured for an additional 24, 48 and 72 h with or without ArtinM (0.25μg/mL). The cells were washed with PBS, fixed and permeabilized with cold absolute methanol, washed with PBS and stained with 0.2% crystal violet (G. Grubler & Company, Berlin, Germany) in 2% ethanol for 10 minutes. After staining, the cells were washed 10 times with PBS, and then lysed with SDS-NaOH (1 mL 10% SDS and 2 mL 1N NaOH). The plate containing the lysates was centrifuged for 5 min at 129 x*g*. The supernatants were transferred to a 96-well plate, and the absorbance measured at 550 nm (Power Wave X Plate Reader; BioTek Instruments, Winooski, VT).

#### Quantification of cells in mitosis

2.5×10^5^ cells/well were plated in 6 well tissue culture plates (Corning Life Sciences) and cultured overnight and then cultured for an additional 20 hours with or without ArtinM (0.25 μg/mL). At this time, 0.05 μg/mL nocodazole (Millipore Sigma) was added to the cultures for 4 hours in order to arrest the cells in prometaphase. The cells were removed from the tissue culture plates with trypsin-EDTA (Thermo Fisher Scientific, Invitrogen), centrifuged for 5 min at 106 x*g*, washed with PBS, and fixed with 70% methanol at -20 °C. After, washing twice with PBS at 4 °C, the cells were treated with 2 mg/mL RNase (Millipore Sigma) at 37 °C for 30 min and then resuspended in PBS containing 50 μg/mL propidium iodide (Millipore Sigma). DNA fluorescence was measured by flow cytometry (*Facsort* using the *CellQuest* program; BD Biosciences, San Jose, CA).

### Statistical analysis

The experimental results were analyzed using non paired Student’s t-test. Differences were considered significant at *p≤0.05.

## Results

In the present study the ability of various concentrations of ArtinM to stimulate RBL-2H3 mast cells *in vitro* and to induce liberation of the different classes of mediators was examined. The mode of mast cell activation was also investigated. Finally, the effect of a low concentration (0.25μg/mL) of ArtinM on mast cell proliferation was characterized.

### 0.25μg/mL ArtinM induces the release of newly synthesized mediators

**Fig 1:**
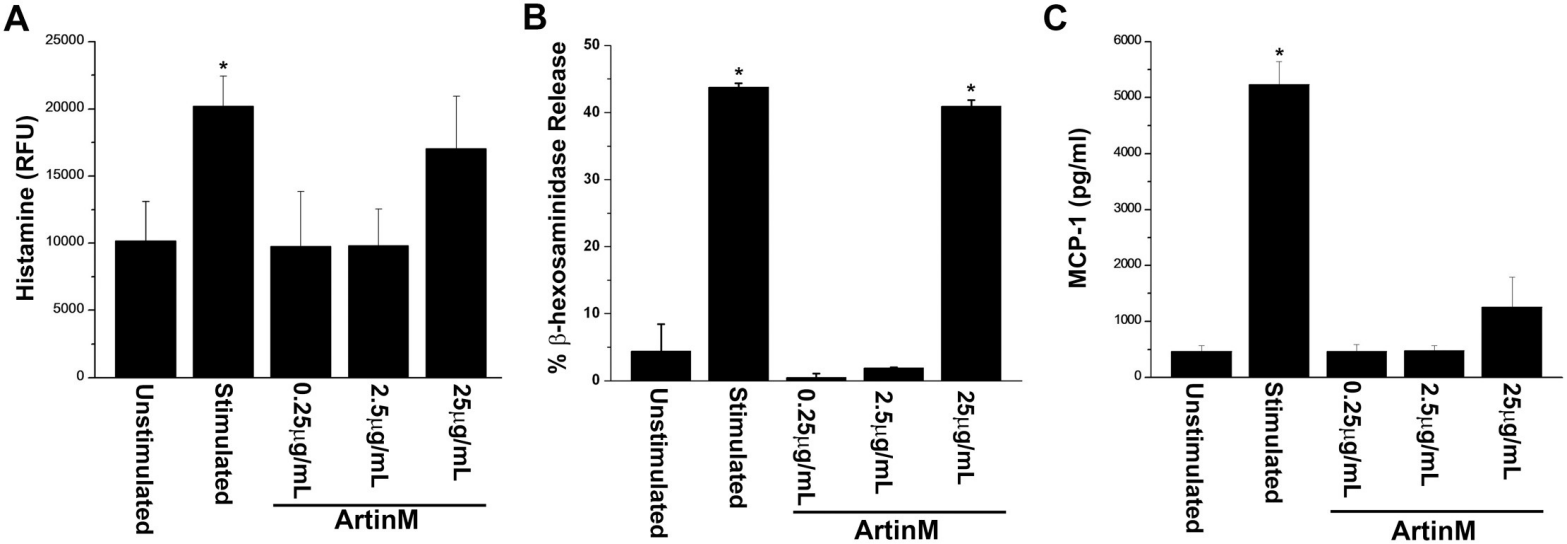
In low concentrations, ArtinM does not degranulate mast cells. RBL-2H3 cells were incubated with 0.25, 2.5 or 25 μg/mL of ArtinM and release of histamine (RFU, relative fluorescence units) (A), β-hexosaminidase (B) and MCP-1(pg/ml) (C) were evaluated. As a positive control, cells were sensitized with IgE anti-TNP and stimulated with DNP_48_-HSA. As a negative control, cells were incubated with media only. The data shown is the average ±SD of 3 independent experiments. * = p≤0.05 when compared to unstimulated cells.

**Fig 2:**
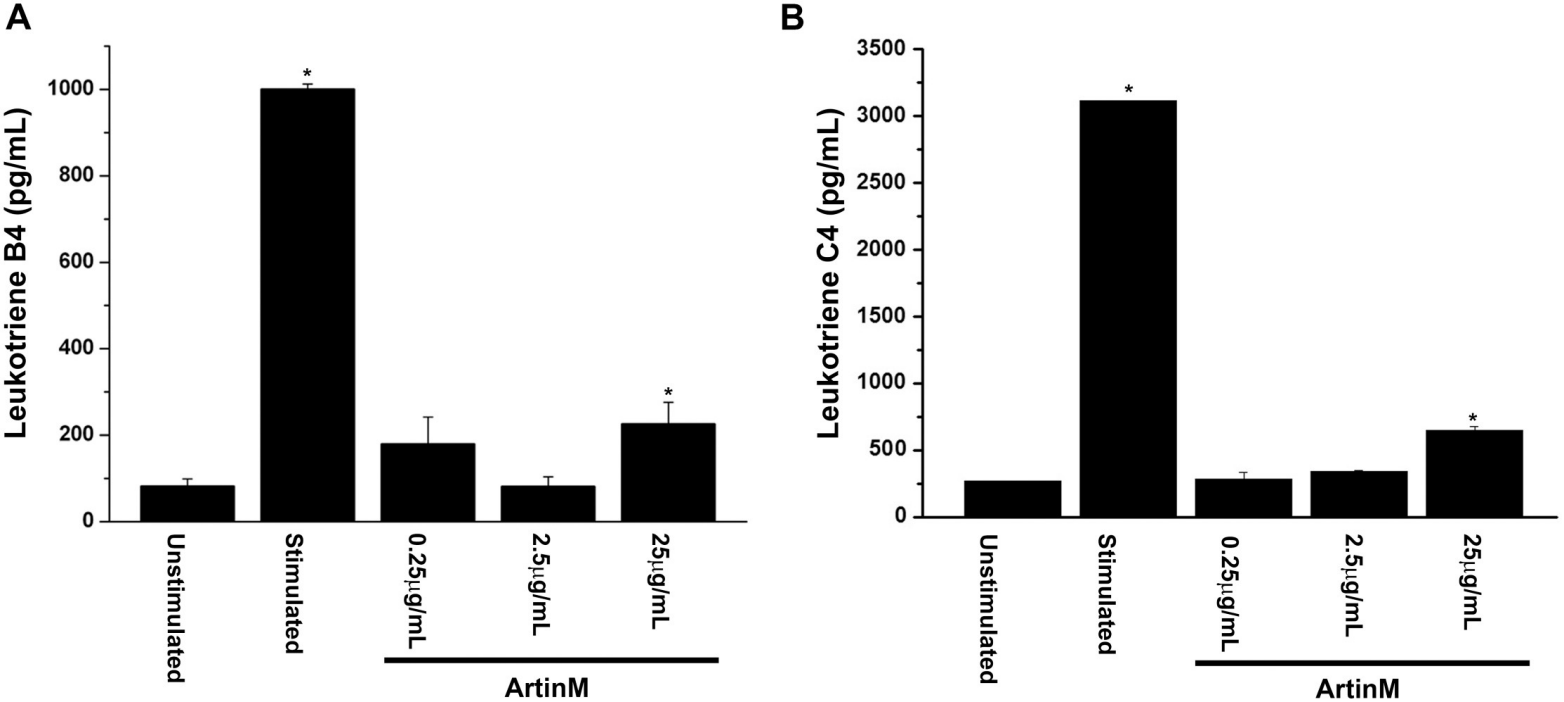
Low concentrations of ArtinM do not induce release of newly formed mediators. RBL-2H3 cells were incubated with 0.25, 2.5 or 25 μg/mL of ArtinM and release of LTB4 (A), and C4 (B) were evaluated. As a positive control, cells were sensitized with IgE anti-TNP and stimulated with DNP_48_-HSA. As a negative control, cells were incubated with media only. The data shown is the average ±SD of 3 independent experiments. * = p≤0.05 when compared to unstimulated cells.

**Fig 3:**
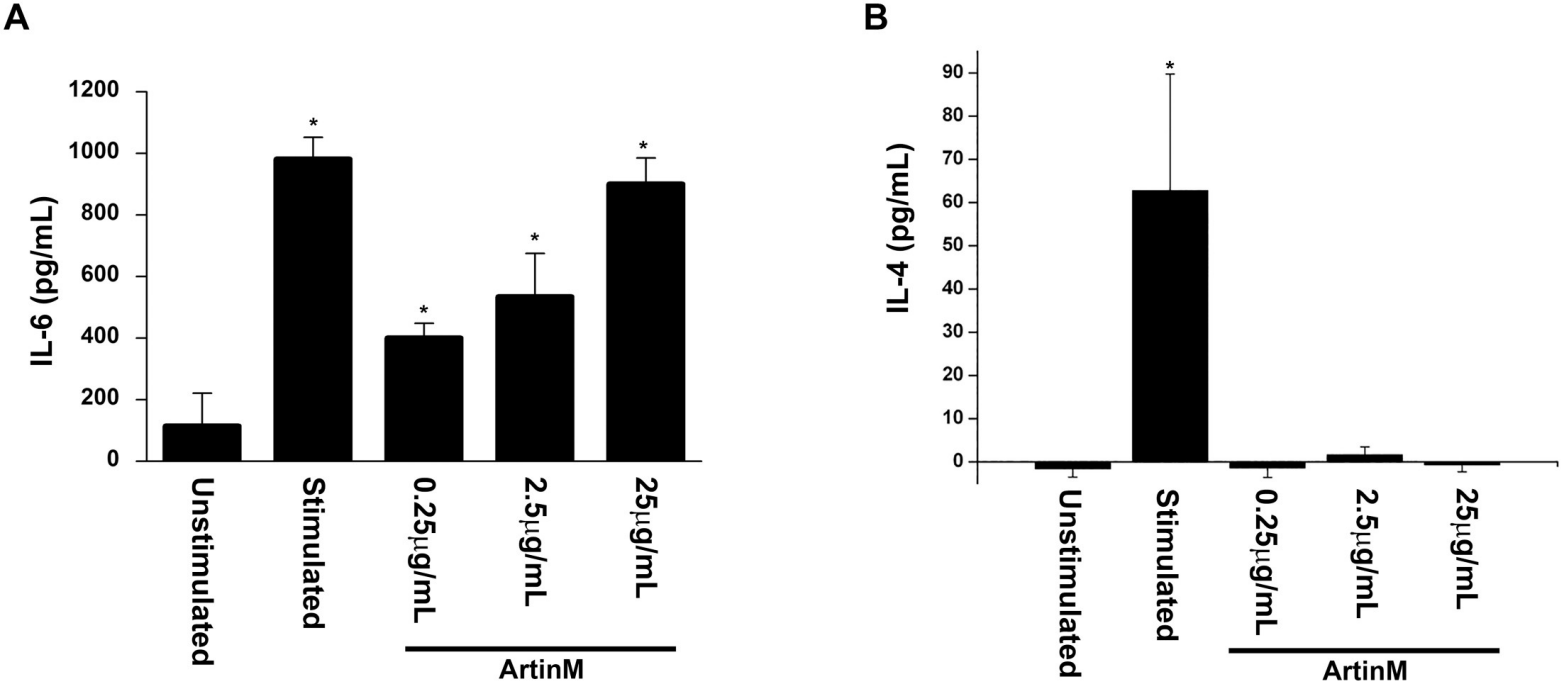
ArtinM stimulates release of IL-6, but not IL-4. RBL-2H3 cells were stimulated with ArtinM (0.25, 2.5 and 25 μg/mL), the supernatants were collected and the concentration of IL-6 (A) and IL-4 (B) were measured by ELISA. As a positive control, cells were sensitized with IgE anti-TNP and stimulated with DNP_48_-HSA. As a negative control, cells were incubated with media only. The data shown is the average ±SD of 3 independent experiments. * = p≤0.05 when compared to unstimulated cells.

In order to examine the effect of a low concentration of ArtinM on mast cell mediator release, RBL-2H3 cells were stimulated with various concentrations of ArtinM (Figs 1–3) and the release of the three classes of mediators (pre-formed, newly formed and newly synthesized) was examined. At 25 μg/mL release of histamine and β-hexosaminidase activity was similar to that seen with antigen stimulation (Fig 1A and 1B). However, at 2.5 μg/mL and 0.25 μg/mL histamine and β-hexosaminidase release was not statistically significant from the unstimulated cells. At 25 μg/mL the release of MCP-1 was slightly higher than unstimulated cells, but this increase was not statistically significant (Fig 1C). At lower concentrations of ArtinM the release of MCP-1 was similar to that of unstimulated cells. Moreover, at 0.25 and 2.5 μg/mLArtinM did not stimulate release of the newly formed mediators LTB4 or LTC4 (Fig 2). At 25 μg/mL, there was a slight but significant release of both LTB4 and C4 when compared to unstimulated cells (Fig 2). However, following stimulation with all concentrations of ArtinM, there was a significant release of IL-6 (Fig 3A), but there was no release of IL-4 (Fig 3B). Notably, the release of IL-6 was dose dependent, with the higher concentrations of ArtinM releasing more IL-6 (Fig 3A).

### ArtinM activates RBL- 2H3 mast cells

#### ArtinM stimulates cell surface ruffling

**Fig 4:**
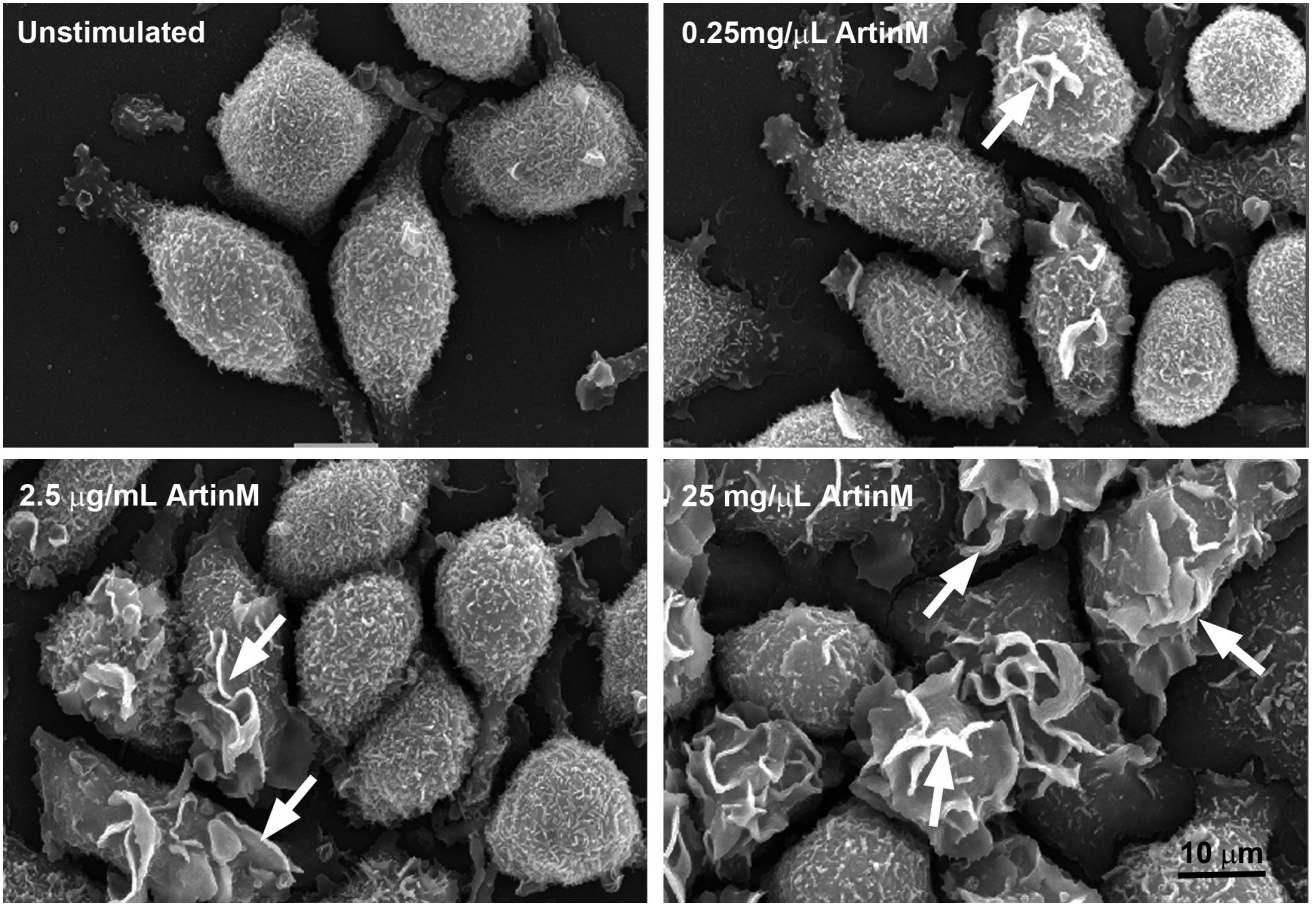
ArtinM induces cell surface ruffles on RBL-2H3 cells. The cell surface of RBL-2H3 cells is covered with short microvilli. Incubation with ArtinM induces ruffling (arrows) of the cell surface in a dose dependent manner.

Activation of RBL-2H3 mast cells is characterized by ruffling of the cell surface [18]. In order to verify mast cell activation by ArtinM, the cells were also evaluated by scanning electron microscopy (Fig 4). The morphological changes observed after incubation with ArtinM correlated with the concentration of ArtinM. The surface of unstimulated cells was covered with fine microvilli, and at 0.25 μg/mL ArtinM there were a few ruffles present on the surface of the RBL-2H3 cells. At 2.5 μg/mL ArtinM, the number of cells with ruffles was increased and at 25 μg/mL ArtinM the majority of cells had prominent ruffles on the cell surface. This ruffling is consistent with activated mast cells.

#### 0.25μg/mL ArtinM stimulates tyrosine phosphorylation

**Fig 5:**
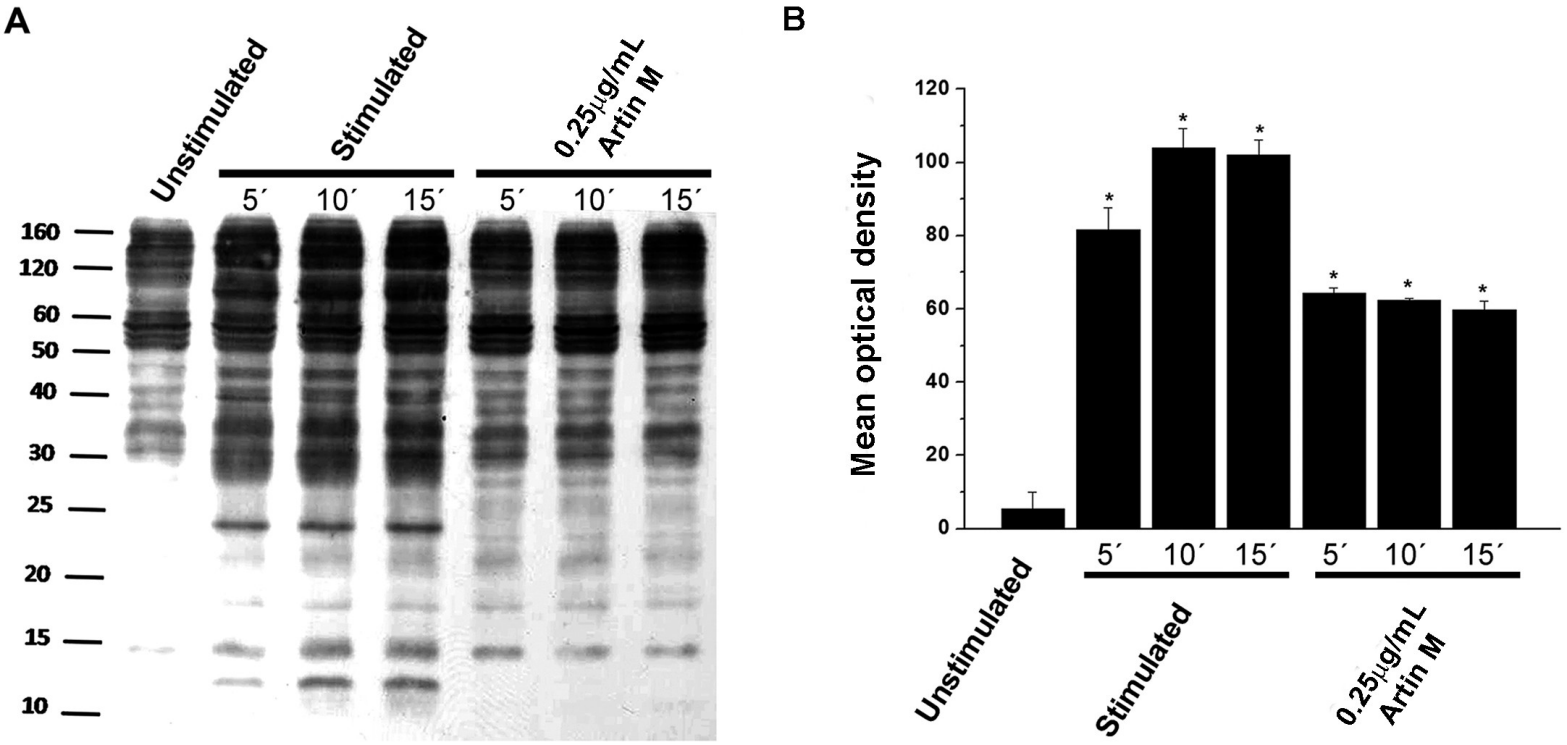
0.25μg/mL of ArtinM stimulates protein tyrosine phosphorylation. RBL-2H3 cells were incubated with 0.25 μg/mL of ArtinM. As a positive control, cells were sensitized with IgE anti-TNP and stimulated with DNP_48_-HSA. As a negative control cells were incubated with media only. (A) Representative Western blot of whole cell lysates were immunoblotted with anti-phosphotyrosine antibody. (B) The mean optical density ±SD of Western blots from three independent experiments is shown. * = p≤0.05 when compared to unstimulated cells.

Since low concentrations of ArtinM activated RBL-2H3 cells without releasing preformed or newly formed mediators, it was of interest to examine one of the initial steps in mast cell activation, protein tyrosine phosphorylation. Exposure of RBL-2H3 cells to 0.25 μg/mL ArtinM induced protein tyrosine phosphorylation, but the extent was less than was seen with antigen stimulation (Fig 5A and 5B). Five minutes after stimulation via FcεRI there was a significant increase in the intensity of the phosphorylated tyrosine bands which was further increased at 10 and 15 minutes. In contrast, the level of phosphorylation seen at 5 min after stimulation with ArtinM was not increased at 10 and 15 min. Thus, although ArtinM does not degranulate mast cells at this low dose, it does activate the cells.

#### ArtinM activates the transcription factor NFκB, but not NFAT

**Fig 6:**
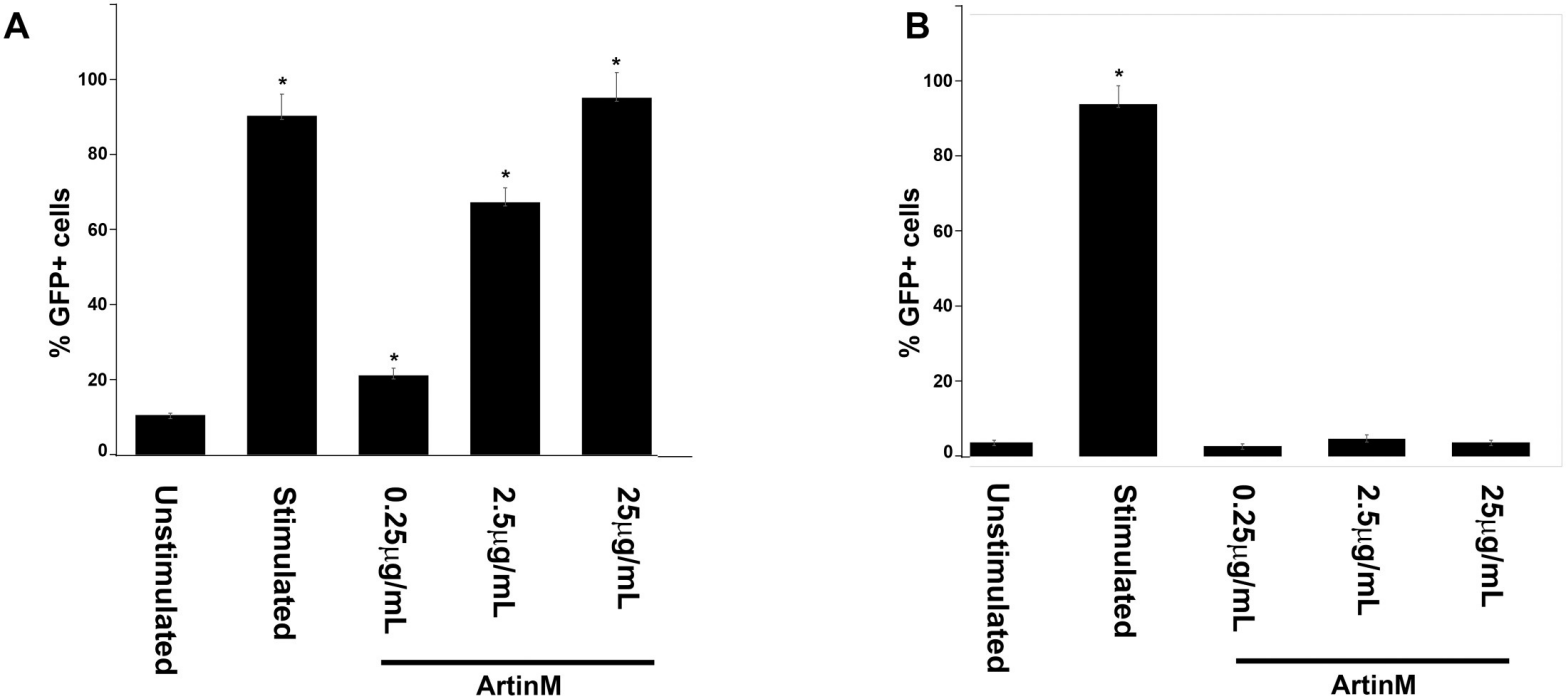
ArtinM activates the transcription factor NFκB, but not NFAT. RBL-2H3 cells transfected with NFκB-GFP (A) or NFAT-GFP (B) were stimulated with 0.25, 2.5 and 25 μg/mL of ArtinM and then examined by flow cytometry for GFP expression. Data is expressed as the % GFP positive cells. Unstimulated and antigen stimulated cells served as negative and positive controls. The data shown is the average ±SD of 3 independent experiments. * = p≤0.05 when compared to unstimulated cells.

At 0.25μg/mL ArtinM induced secretion of IL-6, but not IL-4. Since synthesis of IL-6 is mediated by activation of the transcription factor NFκB, and IL-4 is mediated by NFAT [17], it was of interest to investigate the ability of ArtinM to activate NFκB and NFAT. RBL-2H3 cells transfected with NFκB- or NFAT-GFP were stimulated with ArtinM and then examined by flow cytometry for expression of NFκB-GFP (Fig 6A) and NFAT-GFP (Fig 6B). At all concentrations tested, ArtinM activated only NFκB and not NFAT. The activation of NFkB was dose dependent, similar to that seen for IL-6 release (Fig 3A).

### 0.25μg/mL ArtinM affects the cell cycle and stimulates cell proliferation

**Fig 7:**
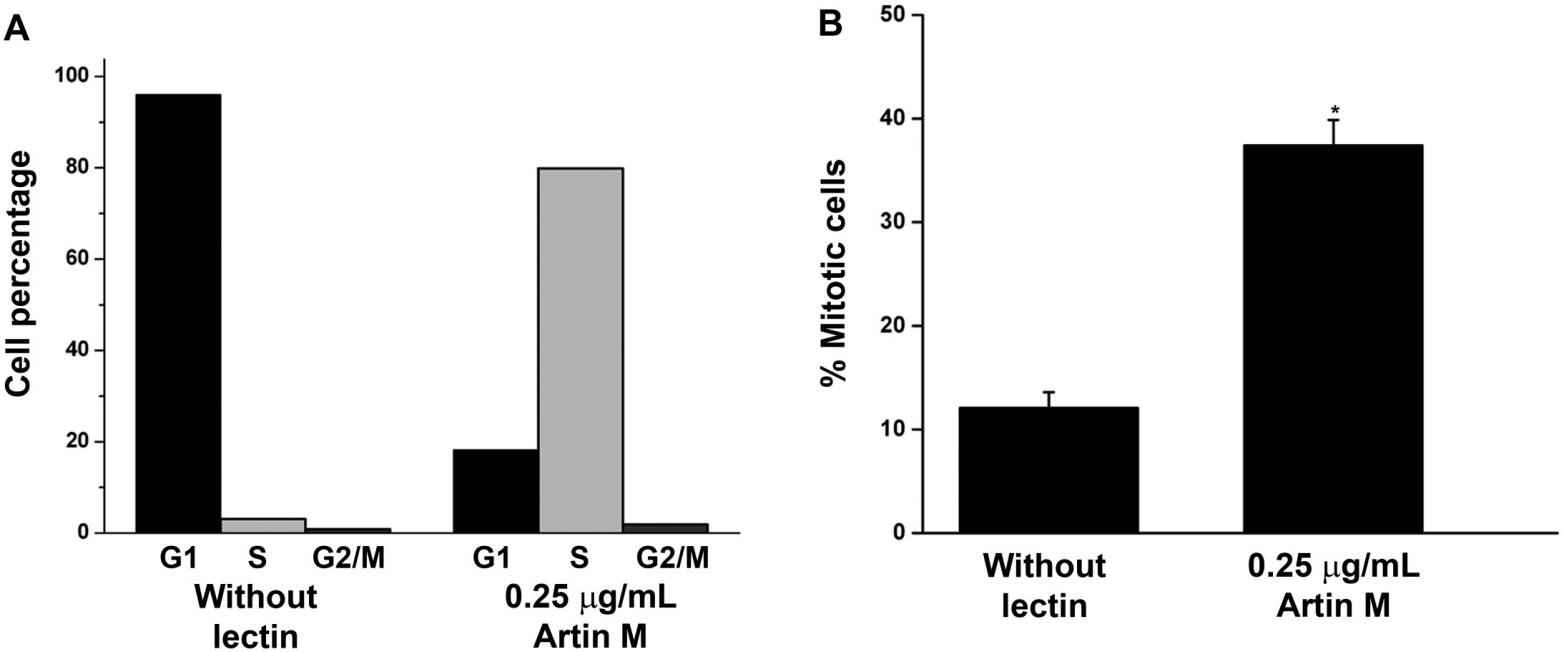
Incubation with 0.25μg/mL ArtinM accelerates the cell cycle. (A) RBL-2H3 cells were synchronized using a double thymidine block. The synchronized cells were incubated with 0.25 μg/mL ArtinM. After 5 h, the cells were stained with propidium iodide. (B) After 5 h, the cells were immunostained with anti-phosphohistone H3, a marker of mitosis, and analyzed by flow cytometry. The data shown is the average ±SD of 3 independent experiments. * = p≤0.05 when compared to cells not incubated with lectin.

Since NFκB is known to interact with the cyclin-dependent kinases which are important regulators of the cell cycle [26], the ability of a low concentration of ArtinM to modulate the cell cycle was investigated. Synchronized RBL-2H3 cells were incubated with or without 0.25 μg/mL ArtinM. In the presence of ArtinM, 5 h after release of the thymidine block, the cell cycle was accelerated with approximately 80% of the cells in S phase. At this same time less than 4% of the untreated cells had entered S phase (Fig 7A). The percentage of untreated cells in S phase was increased beginning at 6 h after release of the thymidine block (S1 Fig). This advancement in the cell cycle was confirmed by an increase in phosphorylated histone H3, which is phosphorylated at the time a cell enters mitosis. Cells were incubated with or without ArtinM for 5 h and immunostained with anti-phosphohistone H3 (Fig 7B). The percentage of ArtinM treated cells that were positive for phosphorylated histone H3 was 40% greater in comparison to the untreated cells.

**Fig 8:**
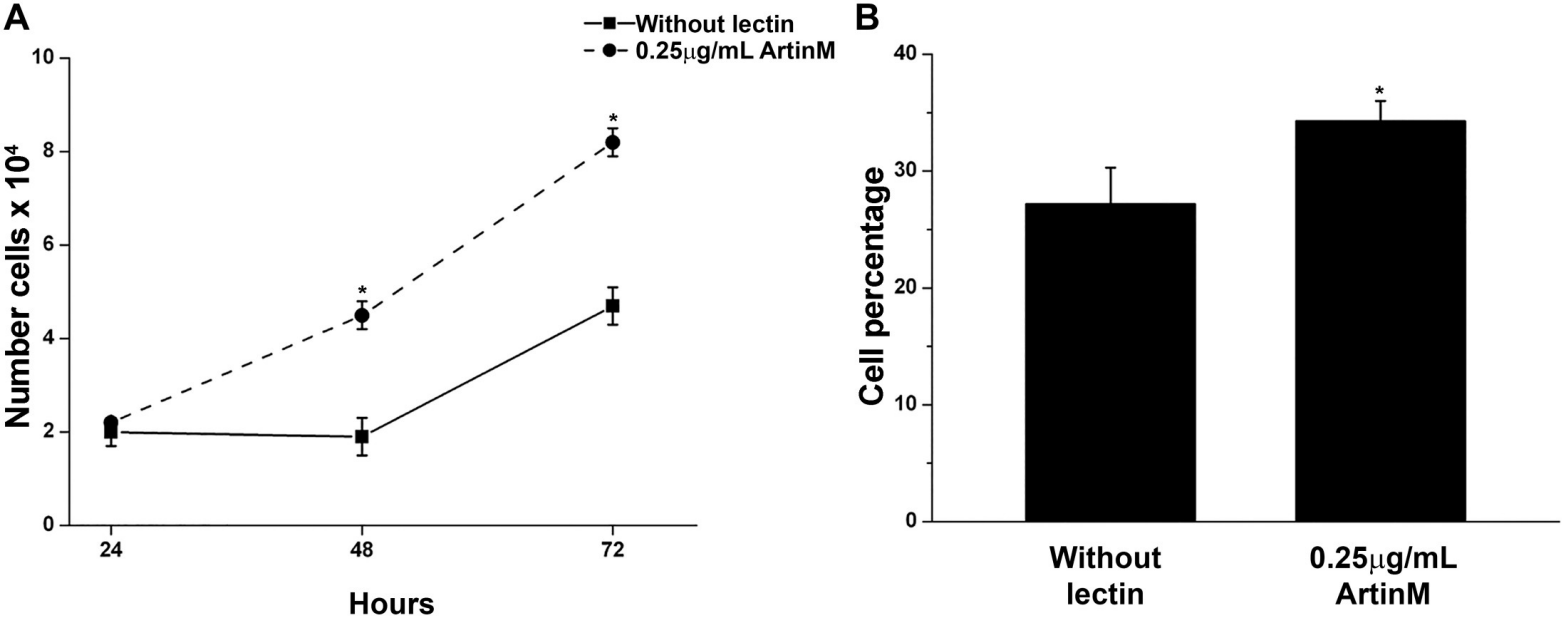
0.25μg/mL ArtinM stimulates the proliferation of RBL-2H3 cells and increases the number of cells in mitosis. (A) RBL-2H3 cells were incubated for 24, 48 and 72 h in the presence or absence of ArtinM and stained with Crystal violet. * = p≤0.05 in relation to cells at the same time point not incubated with lectin. (B) After 20 h in culture, the cells were incubated with nocodazol for 4 h. The cells were then stained with propidium iodide and the percent of cells in mitosis determined by flow cytometry. The data shown is the average ±SD of 3 independent experiments. * = p≤0.05 in relation to cells not incubated with lectin.

The increased number of cells entering S phase after treatment with ArtinM was reflected in an increase in cell proliferation (Fig 8). By 72 h in culture with 0.25 μg/mL ArtinM the number of cells had increased 75% as compared to the untreated controls (Fig 8A). Additionally, the percent of RBL-2H3 cells in mitosis after 24 h of incubation with ArtinM was 30% higher than that seen in the untreated cells (Fig 8B). Therefore, incubation with 0.25μg/mL ArtinM also affected the cell cycle and cell proliferation.

## Discussion

The present study shows that at a low concentration (0.25μg/mL) ArtinM is able to activate RBL-2H3 cells (a mucosal type mast cell) without inducing degranulation. Incubation with 0.25μg/mL ArtinM activated the transcription factor NFκB resulting in the release of the newly synthesized mediator IL-6. Furthermore, as a consequence of cell activation by ArtinM, there was an increase in cell cycle rate, as well as in cell proliferation.

The effects of a low concentration of ArtinM on mast cells seen in this study, verifies the pharmacological potential of ArtinM. The production and release of newly synthesized mediators are closely related to host immune defense and wound healing processes [14, 21]. Interestingly at 0.25μg/mL ArtinM mast cells were not degranulated nor did they release newly formed lipid mediators. Immediately after activation of mast cells via FcεRI, the cells release both pre-formed and newly formed mediators. The release of pre-formed and newly formed lipid mediators contributes to the inflammatory and allergic reactions seen after mast cell activation [27–31]. However, the newly synthesized mediators such as IL-6 [32], released after exposure to a low concentration of ArtinM are important during wound healing by stimulating cell proliferation [33] and neovasculargenesis [34].

ArtinM has the potential to act as an immunomodulator in microbial infections [5, 35, 36]. It exerts a protective effect against *Paracoccidioides brasiliensis* infection by promoting the development of a Th1 immune response. In mice infected with *P*. *brasiliensis*, ArtinM stimulated IL-12 production through a mechanism dependent on TLR2/MyD88 signaling [37, 38]. ArtinM also stimulates human peripheral blood cells to secrete pro-inflammatory Th1-related cytokines [39]. ArtinM was also effective against *Leishmania amazonensis*. Treatment with ArtinM was able to reduce the footpad lesion size, as well the parasite load in *L*. *amazonensis* infected mice [40]. Human neutrophils treated with ArtinM and infected with *Leishmania major*, demonstrated an increased clearance of *L*. *major*, an augmented release of inflammatory cytokines, ROS production, and cell degranulation [41]. Furthermore, the life span of ArtinM-stimulated neutrophils decreased [42]. Additionally, in a murine model of *Toxoplasma gondii* infection ArtinM induced the production of anti-inflammatory cytokines and increased NO levels as well as reducing the parasitic load [43].

*In vivo*, the role of ArtinM as an immunomodulator most likely involves mast cells as well as other immune cells such as neutrophils, macrophages, T cells and B cells. ArtinM activates neutrophils, augmenting their functions such as respiratory burst, phagocytosis of pathogens, secretion of IL-8 and the transcription of the gene for TLR2 [44]. The lectin also stimulates neutrophil migration, most likely by binding to the receptor for IL-8, CXCR2 (CXC chemokine receptor type 2) on the neutrophil surface [1]. ArtinM binds directly to TLR2 N-glycans on macrophages inducing the production of cytokines such as IL-12 and IL-23, which drive the immune response to Th1 and Th17 axis and favor protection against fungal infections [45]. ArtinM also has a direct effect on CD4+ T cells, stimulating IL-17 production [46]. Activation of murine CD4⁺ and CD8⁺ T cells by ArtinM result in increased release of IL-2 and IFN-γ [47]. Furthermore, ArtinM activates murine B cells, increasing IL-17 and IL-12p40 production [48].

Many plant lectins have been shown to affect the cell cycle [49]. Some lectins are able to bind to growth factor receptor glycans on the cell surface. This binding may either inhibit ligand binding to its receptor or may mimic the binding of growth factors to their receptors [50]. In the present study, ArtinM (0.25 μg/mL) induced an increase in RBL-2H3 cell proliferation that was a consequence of an accelerated cell cycle. The mitogenic activity of ArtinM seen in the present study was also seen in epithelial cells of the rabbit cornea treated with ArtinM. In the cornea, an increase in PCNA (Proliferating Cell Nuclear Antigen) and p63 expression was seen. Expression of PCNA and p63 are both important for proliferation and differentiation of the stratified epithelium [6]. In contrast, exposure to high concentrations of ArtinM induced apoptosis in Jurkat T cells [47].

RBL-2H3 mast cells responded differently to high and low concentrations of ArtinM. A similar effect was noted with rat mesenteric mast cells [10]. When mesentery fragments were incubated with 10μg/ml ArtinM, the mast cells did not degranulate. However, when the mesentery fragments were incubated with 200μg/ml 27.4%±2.3% of the mesenteric mast cells were degranulated. In contrast ArtinM degranulated peritoneal mast cells at both concentrations. How mast cells can differentiate the signals required for cytokine production from those that result in secretory granule exocytosis and production of lipid mediators remains unclear [30, 51]. However, there is evidence that mast cells respond differently to varying concentrations of the same stimulus [52, 53]. Also, stimulation through receptors other than FcεRI can result in selective mediator release. For example, activation of Toll-like receptors (TLR) [17, 54] or ganglioside cross-linking [55, 56] on the mast cell surface leads to cytokine production and release in the absence of degranulation. Furthermore, chemokines do not induce mast cell degranulation, but they are able to induce the secretion of newly-synthesized mediators [57].

The α subunit of FcεRI, which is exposed on the cell surface, is highly glycosylated [58]. ArtinM is a homotetramer with 4 carbohydrate recognition domains that is capable of binding to and cross-linking cell surface carbohydrates [59]. It is possible that in low concentrations, the degree of cross-linking may be sufficient to activate mast cells, but insufficient to stimulate degranulation. However, studies by Metzger et al. demonstrated that low concentrations of IgE dimers were sufficient to elicit degranulation in mast cells [60]. Furthermore, as little as 5 ng of antigen results in maximum degranulation in RBL-2H3 cells [61]. The concentration of ArtinM used in the present study is greater than that which would be expected to fully degranulate RBL-2H3 cells. Therefore, at 0.25μg/mL, ArtinM is most likely exerting its effects by binding to other cell surface components. There are numerous other ligands that can activate mast cells by binding cell surface components other than FcεRI [62]. The binding of many of these ligands such as SCF (stem cell factor), CCL3 (CC-chemokine ligand 3) and PAMPs (pathogen-associated molecular patterns) results in selective mediator release without degranulation [63]. Furthermore, cross-linking GD1b derived gangliosides on the surface of RBL-2H3 cells causes the release of newly formed and newly synthesized mediators without degranulation [55].

In the present study it was observed that a low concentration of ArtinM (0.25 μg/mL) induced NFκB activation. NF-κB proteins are a major family of transcription factors involved in control of the immune response [64–67]. This family consists of 5 members: NF-*κ*B1 (p50) NF-*κ*B2 (p52), RelA (p65), RelB and c-Rel, which bind DNA as homo- or heterodimers. The predominant active NF-*κ*B dimer in mast cells, is a p50/RelA heterodimer [68]. Mast cell activation through cross-linking FcεRI or cell surface gangliosides, ligand binding to Toll-like receptors (TLR) or the substance P receptor induces NFkB activation resulting in the release of IL-6 and TNF- α [15, 69–71]. Furthermore, in mast cells the Bcl10/Malt1 complex specifically uncouples the pathway for degranulation from that of cytokine production [52, 66], thus supporting the current findings that ArtinM can induce cytokine production without stimulating degranulation.

## Conclusions

The present investigation demonstrated that a low dose (0.25μg/mL) of ArtinM was able to induce mast cell activation, NFκB activation with consequent IL-6 release and enhanced cell cycle and proliferation rates without inducing degranulation and lipid mediator release. Taken together with the effects previously described for ArtinM, these results suggest a potential role for ArtinM as a therapeutic tool in modulating immune responses.

## Supporting information

S1 Fig ArtinM accelerates the cell cycle. (A) When cells were cultivated without ArtinM, the peak of cells in S phase occurred at 6 hours after release of the thymidine block. (B) In the presence of ArtinM, the cell cycle was accelerated with approximately 80% of the cells in S phase 5 h after release of the thymidine block.
(PDF)

S2 Fig Fig 5A original gel. (PDF)
