# Supplementary material for: The lectin ArtinM activates RBL-2H3 mast cells without inducing degranulation
Source: PLoS One. 2020 Mar 24;15(3):e0230633. doi: 10.1371/journal.pone.0230633 (PMC7092976; doi:10.1371/journal.pone.0230633)
Supplement: S1 Fig — (A) When cells were cultivated without ArtinM, the peak of cells in S phase occurred at 6 hours after release of the thymidine block. (B) In the presence of ArtinM, the cell cycle was accelerated with approximately 80% of the cells in S phase 5 h after release of the thymidine block. (PDF) [file pone.0230633.s001.pdf]

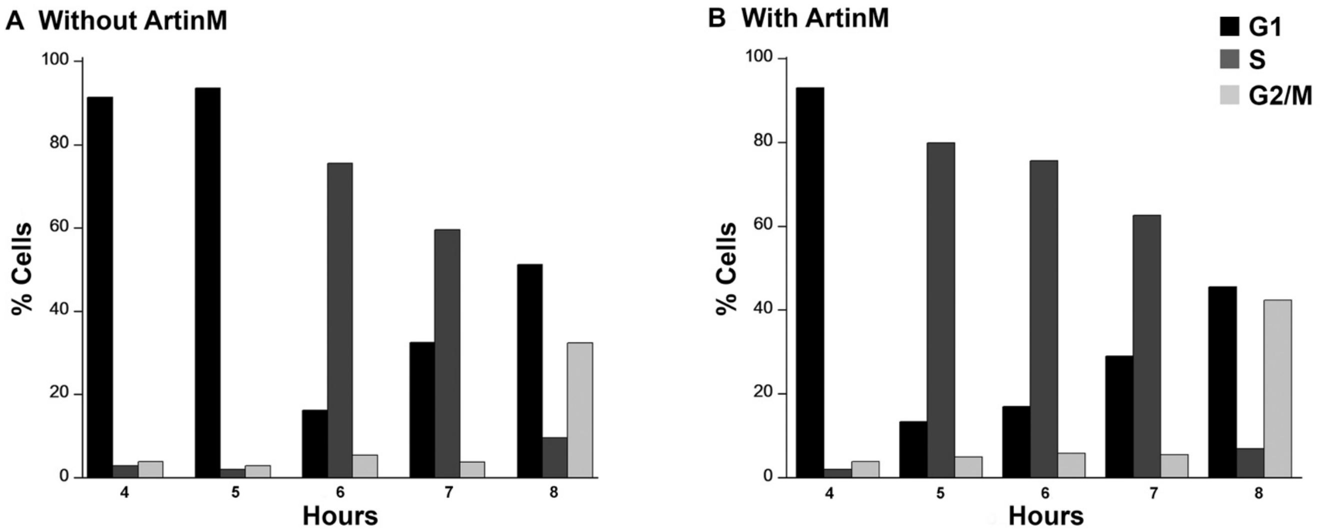

S1 Figure. ArtinM accelerates the cell cycle. (A) When cells were cultivated without ArtinM, the peak of cells in S phase occurred at 6 hours after release of the thymidine block. (B) In the presence of ArtinM, the cell cycle was accelerated with approximately 80% of the cells in S phase 5 h after release of the thymidine block.
