# Supplementary figures and images for: The lectin ArtinM activates RBL-2H3 mast cells without inducing degranulation
Source: PLoS One. 2020 Mar 24;15(3):e0230633. doi: 10.1371/journal.pone.0230633 (PMC7092976; doi:10.1371/journal.pone.0230633)

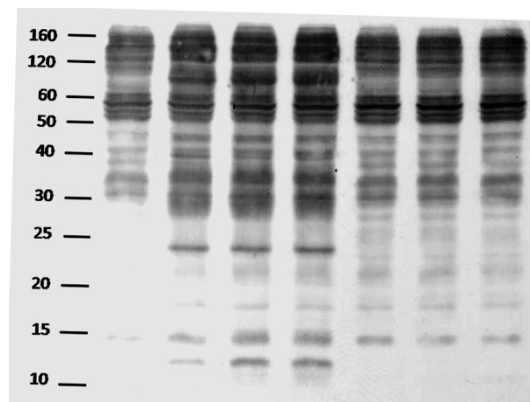

**S2 Gel. Original Gel Figure 5A**

Supplement: S2 Fig — (PDF) [file pone.0230633.s002.pdf]
